# First evidence of ranunculids in Early Cretaceous tropics

**DOI:** 10.1038/s41598-022-07920-y

**Published:** 2022-03-23

**Authors:** William Vieira Gobo, Lutz Kunzmann, Roberto Iannuzzi, Julien B. Bachelier, Clément Coiffard

**Affiliations:** 1 Departamento de Paleontologia e Estratigrafia, Universidade Federal do Rio Grande do Sul (UFRGS), Av. Bento Gonçalves 9500, 91509-900 Porto Alegre, Rio Grande do Sul Brazil; 2 Abteilung Museum für Mineralogie und Geologie, Senckenberg Naturhistorische Sammlungen Dresden, Königsbrücker Landstrasse. 159, 01109 Dresden, Germany; 3 Structural and Functional Plant Diversity Group, Institute of Biology, Freie Universität Berlin, Altensteinstrasse 6, 14195 Berlin, Germany

**Keywords:** Plant sciences, Evolution, Palaeontology, Taxonomy

## Abstract

Early Cretaceous floras containing angiosperms were described from several geographic areas, nearly from the Arctic to the Antarctic, and are crucial to understand their evolution and radiation. However, most of these records come from northern mid-latitudes whereas those of lower paleolatitude areas, such as the Crato Fossil Lagerstätte in NE Brazil, are less studied. Here, we describe from this region of northern Gondwanan origin, two fossil-species of eudicots belonging to a new extinct genus *Santaniella* gen. nov. Together with several vegetative axes and leaves, anatomically well-preserved fruits with seeds and persistent perianth-like organs allowed us to reconstruct its potential affinities with ranunculids, and presumably Ranunculaceae. Previous records putatively assigned to Ranunculales are all from mid-latitudes, and their first unequivocal occurrence in a low-latitude area supports further the hypothesis of a widespread radiation of the earliest diverging eudicot lineage by this early age.

## Introduction

The apparently rapid diversification of flowering plants in fossil records was famously referred to as an “abominable mystery” and “most perplexing phenomenon” by Darwin, who often advocated for a gradual evolution^1^. Nowadays, the fossil record demonstrates that the origin and early radiation of angiosperms was indeed less abrupt than previously thought^2^. Based on extensive evidence, we know now that the first flowering plants appeared at low-latitude areas in Northern Gondwana in the Early Cretaceous (ca. 135 Ma), and then they gradually began to disperse to mid- and high-latitudes in both hemispheres throughout the Cretaceous^3^. However, the inherently incomplete fossil record still hinders a precise reconstruction of the early evolutionary history of angiosperms and diversification of the main extant lineages, such as the eudicots.

Eudicots are an extremely diverse monophyletic group encompassing about three quarters of all living flowering plants^4^. They comprise a grade of early-diverging lineages, i.e., Ranunculales, Proteales, Trochodendrales and Buxales, that are successively sisters to the rest^5^. However, while the resolution of their phylogenetic relationships and systematic affinities have been constantly improved over the past 30 years, their origin and early evolutionary history are still elusive because our knowledge is based mostly on dispersed pollen and mesofossils, whereas the macrofossil record is scarce and mostly fragmented^2,6^.

The oldest record of eudicots extends back to at least the middle Early Cretaceous mainly in low-latitude areas and based mostly on their distinct tricolpate pollen grains, found in sedimentary rocks since the late Barremian (ca. 125 Ma)^2^. In addition, their presence in the Barremian and Aptian is strongly supported not only by abundant dispersed pollen grains, but also by the presence of in situ pollen in mesofossils including isolated stamens and carpels^2,7^. In contrast, unambiguous pre-Albian (before 113 Ma) macrofossils are relatively rare, whereas the diversity of eudicots seems to increase mainly during the Albian and Cenomanian (113–94 Ma) until they eventually start to dominate most plant fossil assemblages from the Late Cretaceous (ca. 100 Ma) onwards^2^.

The accelerated evolution and diversification of basal eudicots are expected during the late Barremian and early Aptian (ca. 125 Ma)^8^. In early-diverging eudicots, Ranunculales are sisters to all other lineages and like Proteales, Trochodendrales and Buxales represented by a handful of fossil records in the Early Cretaceous, most of which are not affiliated to extant families^2,5^. To date, molecular estimates using fossil-based calibrations indicate that the emergence of the crown-group of Ranunculales started only around 123 to 112 Ma^4,9,10^ and the origin of the crown group of Ranunculaceae at around 108 Ma^11^. In addition, the few undisputed macrofossils with ranunculid/basal eudicot affinities are derived from mid-paleolatitudes in the Northern Hemisphere^12–24^, whereas the low-paleolatitudes, that may have been the cradle for eudicots, are still mostly “*terra incognita*”.

Northern Gondwanan floras (e.g., Crato Formation flora), which represent the paleoequatorial vegetation during the Early Cretaceous, provide important insights in the evolution and early radiation of angiosperms and their biology. In the Crato Formation from Brazil, one-third of the total angiosperm pollen record is represented by tricolpate grains^25^, but no unquestionable eudicot megafossil was described yet (see discussion^6^).

Here, we report for the first time specimens whose exceptional preservation of vegetative and reproductive parts supports affinities with extant Ranunculales, and thus, also represent the most complete ranunculid fossils described to date from the Early Cretaceous tropics.

### Details of specimens

#### Geological setting and age

The study specimens are from the Crato limestones termed Pedra Cariri within a lithostratigraphic unit known as the Crato Formation, belonging to the Santana Group in the Jurassic-Cretaceous Araripe Basin (NE Brazil)^26^. They have been gathered at open cast pits close to the town of Nova Olinda (Ceará) by local workers who excavate the laminated lacustrine carbonate rock as cut and ornamental stone. Recent dating of other Santana Group formations assumed an early Aptian or even late Barremian age for the Crato Formation^27,28^. However, this age is not consensual since former estimations suggested a late Aptian/early Albian age of this geologic unit (^29^ and references therein). Due to the diversity, preservation and abundance of biota, as well as its paleogeographic and temporal context, the Crato limestones contain one of the most important Early Cretaceous fossil lagerstätten in Gondwana^30^.

#### Associated plant taxa

In the Crato Formation flora, the numbers of taxa range from more than 40 based on macrofossils and more than 150 based on palynomorphs of spore and seed-bearing plants, and not all were formally described yet^30,31^. In addition, the angiosperm component of the Crato Formation flora is diverse both taxonomically and ecologically^32^, and includes several fossil-taxa with clear affinities with early-diverging extant lineages (ANA grade^33,34^) or mesangiosperms (e.g., magnoliids^35–38^, monocots^32,39^). Several early eudicot taxa were reported earlier but none had been described yet^30^.

Angiospermae Lindley (P.D. Cantino & M.J. Donoghue)

Eudicotyledoneae M.J. Donoghue, J.A. Doyle & P.D. Cantino

Order Ranunculales Juss. ex Berchtold & J. Presl

Family cf. Ranunculaceae Juss.

*Santaniella* Gobo, Coiffard, Bachelier, L. Kunzmann et Iannuzzi gen. nov.

#### Generic diagnosis

Angiosperm with herbaceous to suffruticose growth habit. Thin elongated axes with leaves ranging from sessile and simple distally to increasingly longer, larger, and more divided and lobed towards the base. Proximal leaves pedately compound with typical bifurcation into two (type II) or more leaflets (type III). Leaf and leaflets unlobed to lobed with untoothed margin. Petiole (and petiolules) with marginal attachment. Primary venation pinnate and secondary craspedodromous, branching dichotomously and merging into a perimarginal vein. Anastomoses rare. Fruit a solitary and terminal follicetum derived from an apocarpous, superior gynoecium with 2 to up to at least 11 multiovulate plicate carpels developing into stipitate, reflexed and pendulous follicles, born on a short, flattened to slightly concave receptacle typically surrounded by persistent perianth-like organs. Fruitlet comprises at least up to 9 seeds arranged in a row along a median longitudinal placenta on the abaxial side.

#### Etymology

The genus name is derived from the lithostratigraphic unit known as the Santana Group where the studied specimens come from.

### Plant Fossil Names Registry Number

PFN002910 (for new genus)

### Type species

*Santaniella lobata* sp. nov.

#### Remarks

**Figure 1:**
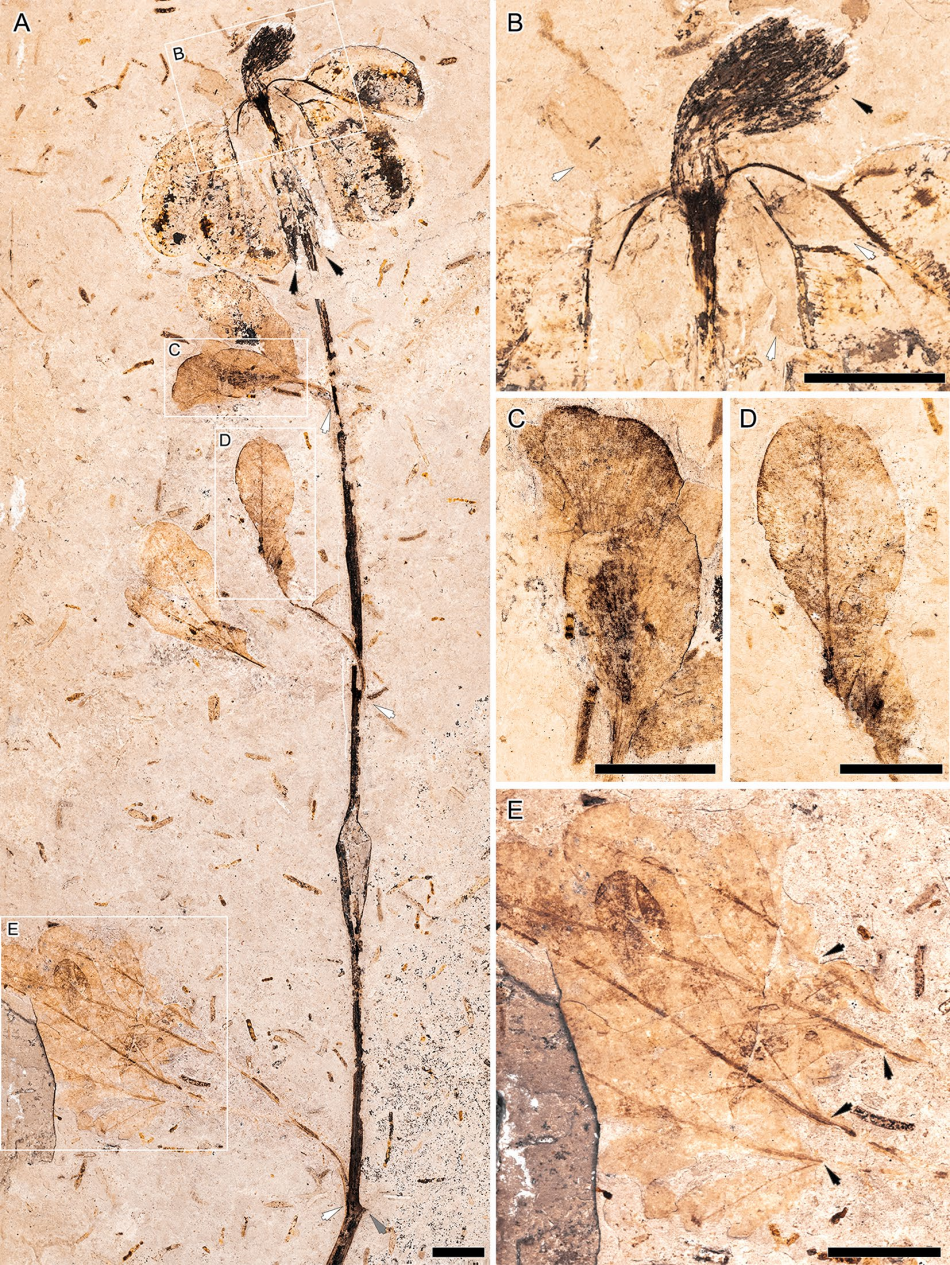
*Santaniella lobata* gen. nov. et sp. nov. (**A–E**) holotype (MB. Pb. 1998/691). (**A**) general view of holotype showing several leaves and a terminal aggregate fruit, with 5–6 stipitate multi-seeded follicles (white arrows point to leaf attachment, gray arrow to a putative opposite leaf, and black arrows to pair of distal most leaves); (**B**) close-up on follicles attachment to the receptacle, surrounded by 3 outer reflexed (white arrows) and several inner erect and laciniate persistent perianth-like organs (black arrow); (**C**–**E**) close-up on leaves showing a gradual acropetal reduction in organization and size from (**E**) to (**C**); (**C**, **D**) leaf type II. (**E**) leaf type III with four lobed leaflets (black arrows point to primary veins). Scale bars = 1 cm.

**Figure 2:**
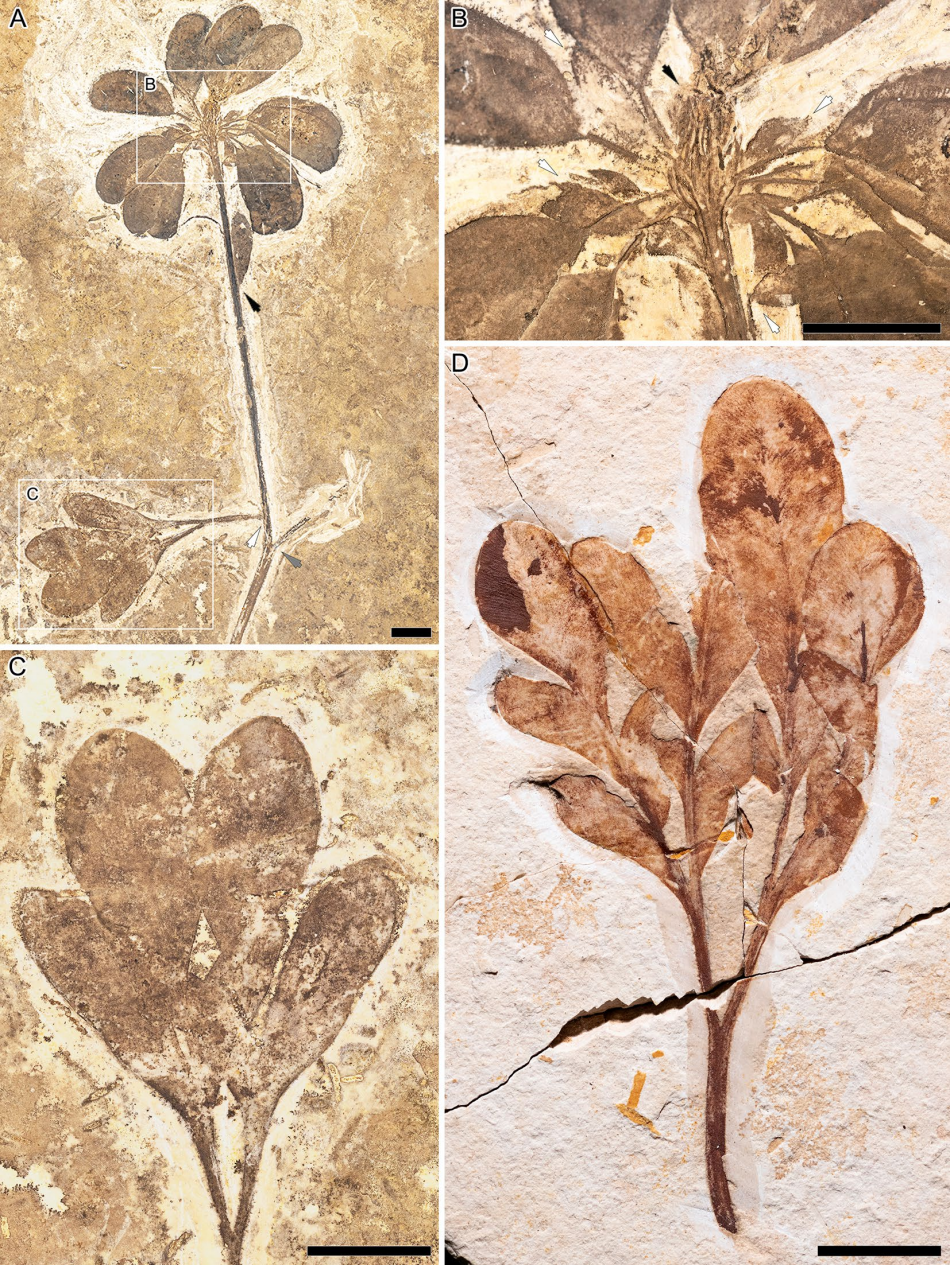
*Santaniella lobata* gen. nov. et sp. nov. (**A**–**C**) paratype (MB. Pb. 1997/1347). (**A**) general view of paratype showing leaves and a terminal aggregate fruit with 10–11 stipitate follicles (black arrow points to leaf attachment of the distalmost leaf, white arrow to leaf attachment of the basalmost leaf, and gray arrow to a putative sub-opposite leaf); (**B**) close-up on follicles surrounded by 4 outer (white arrows) and several innermost erect and more or less laciniate (black arrow) persistent perianth-like organs; (**C**) close-up on leaf type II with two trilobed leaflets; (**D**) leaf type III (paratype: LP UFC CRT 1559a). Scale bars: 1 cm.

**Figure 3:**
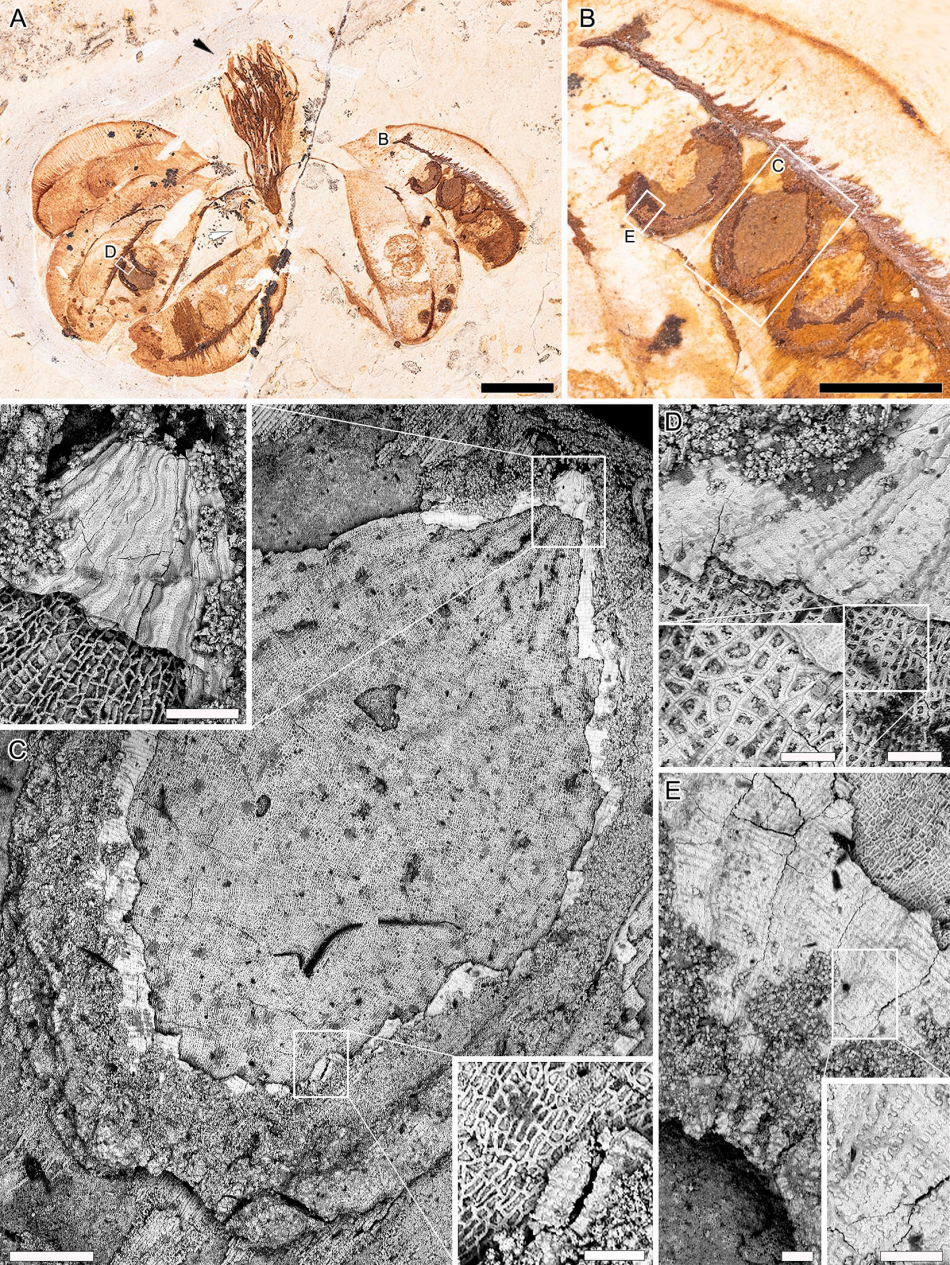
*Santaniella lobata* gen. nov. et sp. nov. (**A**–**E**) paratype (MB. Pb. 1997/1562); (**A**) fruit with one outer (white arrow) and several inner (black arrow) erect and laciniate persistent perianth-like organs surrounding the stalks of 6 stipitate, reflexed and pendulous follicles; (**B**) close-up of 3(-4) permineralized seeds; (**C**–**E**) SEM images of seeds; (**C**) whole seed with inserts showing higher magnification of the outer and inner layers of seed coat in the micropylar and chalazal region. Note the jigsaw puzzle-shaped surface cells in chalazal region; (**D**) close-up of the micropylar region with spiral structures inside the cells of the innermost layer of the seed coat; **(E)** close-up of the chalazal region with jigsaw puzzle outer layer of the seedcoat. Scale bars: (**A**, **B**) = 1 cm; (**C**) = 500 μm, (**C**) close-ups = 100 μm; (**D**) = 100 μm, (**D**) close-up = 50 μm; (**E**) and (**E**) close-up = 100 μm.

**Figure 4:**
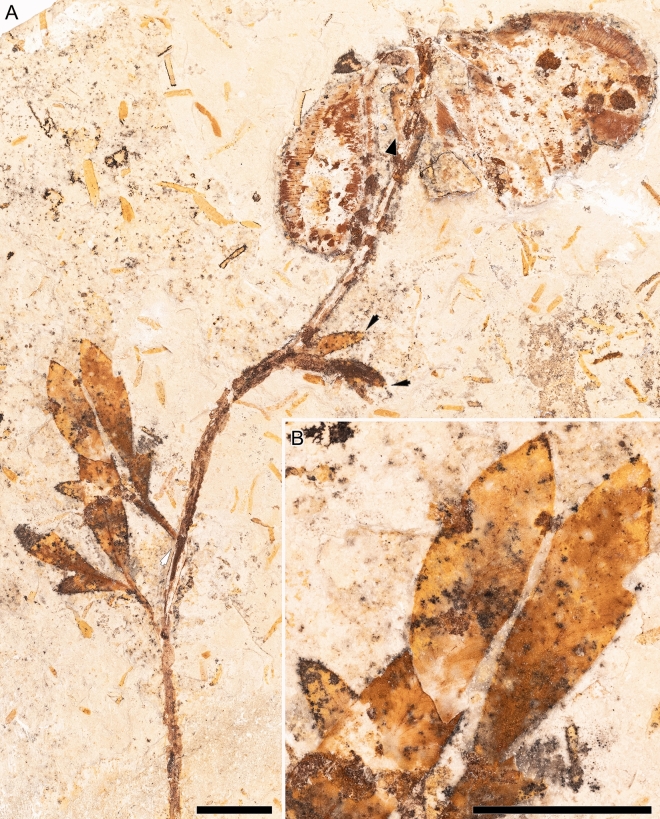
*Santaniella acuta* gen. nov. et sp. nov. (**A**, **B**) holotype (MB. Pb. 2000/71). (**A**) general view of holotype showing four leaves (white arrow points to leaf attachment of the second proximal leaf of type II and black arrows to distalmost leaves of type I), and a terminal aggregate fruit surrounded by only one persistent perianth-like organ (black triangle). (**B**) close-up on leaf showing details of venation and apices. Scale bars: 1 cm.

**Figure 5:**
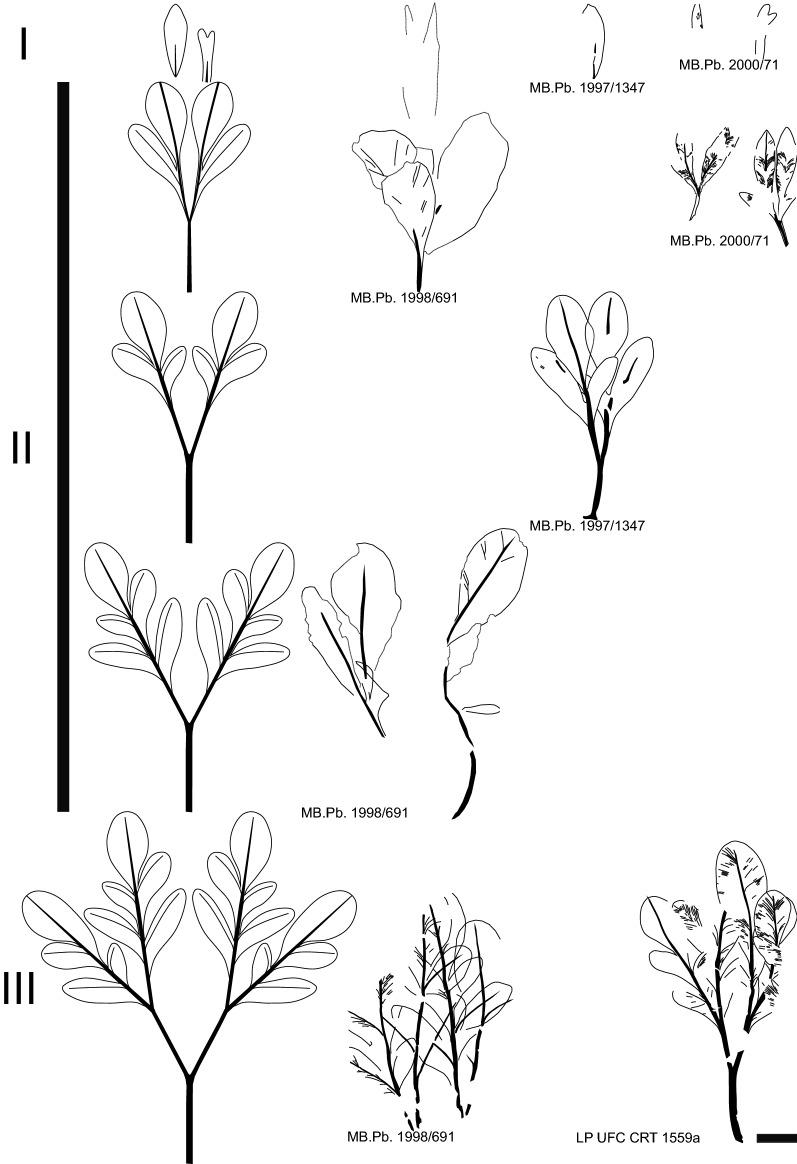
Line drawings of leaf type reconstructions of *Santaniella* gen. nov. and tentative fossil assignments. *S. lobata* = MB. Pb. 1998/691, MB. Pb. 1997/1347, and LP UFC CRT 1559a; *S. acuta* = MB. Pb. 2000/71. I, leaf type I: sessile and simple, typically last below the fruit (MB. Pb. 1998/691, MB. Pb. 1997/1347, MB. Pb. 2000/71); II, leaf type II: bifurcate, and can be interpreted as a single leaf with sessile leaflets (MB. Pb. 1998/691), or pedately compound leaves with one bifurcation into two sessile leaflets (MB. Pb. 2000/71). Larger leaves are more clearly pedately compound with distinctly petiolulate and trilobed leaflets (MB. Pb. 1997/1347). Other leaf fragments (MB. Pb. 1998/691) can be interpreted as a single leaf with two leaflets. III, leaf type III: pedately compound leaves with successive bifurcations forming four odd-pinnatisect leaflets (MB. Pb. 1998/691 and LP UFC CRT 1559a). Scale bars = 1 cm.

Vegetative and reproductive materials of the two fossil-species within the fossil-genus are preserved either as impressions, permineralizations with iron oxide, and/or coalifications, and beside the designation of the holotypes (Figs. 1, 4), four paratypes (Figs. 2, 3, Supplementary Fig. 1) are selected because they exhibit vegetative and/or reproductive diagnostic characters that are not always clearly visible in the holotype of *S. lobata*. All three leaf type reconstructions and potentially corresponding fossils are illustrated in Fig. 5.

*Santaniella lobata* Gobo, Coiffard, Bachelier, L.Kunzmann et Iannuzzi sp. nov.

#### Specific diagnosis

Leaves nanophylls and simple to microphylls with at least two to four leaflets. Leaflets usually petiolulate, pinnately lobed and odd-pinnatisect, with acute and decurrent base and obtuse and rounded lobes. Lamina chartaceous. Fruit surrounded by numerous persistent perianth-like organs, typically erect and distally laciniate.

#### Etymology

The specific epithet refers to the lobed nature of the leaflets.

#### Holotype

MB.Pb. 1998/691 (repository: Museum für Naturkunde—Leibniz Institute for Evolution and Biodiversity Science, Berlin, Germany), Fig. 1.

#### Paratypes

MB.Pb. 1997/1347 (Fig. 2A–C), MB.Pb. 1997/1562 (Fig. 3), and MB.Pb 1997/1348 (Supplementary Fig. 1) (repository: Museum für Naturkunde—Leibniz Institute for Evolution and Biodiversity Science, Berlin, Germany); LP UFC CRT 1559a (Fig. 2D) (repository: Federal University of Ceará, Department of Geology, Fortaleza, Brazil).

### Plant Fossil Names Registry Number

PFN002911 (for new species)

#### Type horizon and locality

Open cast pit(s) southwest of Nova Olinda, Ceará, NE Brazil. Lower Cretaceous (uppermost Barremian?/ Aptian), C6 limestone horizon, Crato Formation, Santana Group, Araripe Basin.

#### Description and remarks

The holotype (MB.Pb. 1998/691) consists of a leafy axis with a terminal fruit (Fig. 1). The axis is about 230 mm long and 2.6 mm wide, and bears several leaves showing a gradual acropetal reduction in both structure and size (Fig. 1A, C–E). The lowest and most proximal leaf is pedately compound and of type III (Fig. 5), with four overlapping lobed and petiolulate leaflets (Fig. 1E), which is better preserved in the second paratype (see below, Fig. 2D). The petiole is 21 mm long and 1.1 mm wide, and the leaf blade is microphyllous, longer and wider than 40 mm. The second and more distal leaf fragments are poorly preserved and can be interpreted as two leaflets of a single leaf of type II, or a pair of leaves of the same type in which the bifurcation is not clearly visible (Figs. 1A,D, 5). The petiole is about 28 mm long and 1.2 mm wide, and the attached leaf fragment is 72 mm long. Individual lobes are 28–35 mm long and 8.5–15 mm wide. The third set of leaf fragments is also poorly preserved and can be interpreted either as a single leaf with two sessile leaflets or a pair of leaves type II (Figs. 1A,C, 5). The petiole is 7.8 mm long and 1.3 mm wide and the attached leaf fragments are 28–31 mm long and 11–13 mm wide. Below the fruit, the distalmost leaves are simple and of type I (Figs. 1A, 5). They are nanophylls, 24 mm long and 2.8 mm wide. L/W ratio is ca. 8:1 and the overall shape is unclear. We do not exclude that they are subtending bracts or a pair of bracteoles.

The aggregate fruit comprises 5–6 stipitate and pendulous follicles. They are 23–30 mm long and 11–12 mm wide, with an 8–8.5 mm long stalk (Fig. 1A). The follicles are connected to a receptacle surrounded by two types of persistent perianth-like organs. The three outermost organs are obovate, with an entire margin and ca. 10 mm long and 6 mm wide (Fig. 1B). Their original organization and number are uncertain but some parts indicate that they were free and reflexed. In contrast, the innermost organs are erect and laciniate (Fig. 1B).

The first paratype (MB.Pb. 1997/1347) is also a leafy axis with a fruit (Fig. 2A). The axis is about 137 mm long and 2.6 mm wide. The most proximal leaf is also pedately compound and of type II (Fig. 5), with two overlapping 3-lobed petiolulate leaflets (Fig. 2C). The petiole is 14 mm long and 1.6 mm wide, and the leaf blade is microphyllous and 36 mm long by ca. 40 mm wide. L/W ratio is about 1:1 and the overall leaf shape is ovate and symmetrical. The leaflets are microphyllous and 36 mm long and 22 mm wide. Their base is acute and decurrent, whereas their lobes are obtuse and rounded. The most distal leaf is simple nanophyll of type I (Figs. 2A, 5) and 18 mm long and 5.2 mm wide. L/W ratio is about 3:1 and the lamina shape is obovate. It is unclear, however, whether there was a second (sub)opposite proximal leaf, and we do not exclude that the distal one is a subtending bract, or one of two bracteoles (Fig. 2A).

The fruit of the first paratype is an aggregate and comprises 10–11 follicles. They are 21–25 mm long and 10–11 mm wide, with a 6–9 mm long stalk (Fig. 2A, B). They are also surrounded at the base by two types of persistent perianth-like organs, comprising 4 outer organs that are very similar to the distalmost leaf, and inner organs with a large base and a more or less laciniate apex (Fig. 2B).

The second paratype (LP UFC CRT 1559a) is a single, well-preserved complete pedately compound leaf of type III (Fig. 5) showing successive dichotomous bifurcations resulting into four lobed asymmetrical leaflets with untoothed margins (Fig. 2D). The complete leaf is reconstructed as being 70 mm long and ca. 50 mm wide. The petiole is about 17 mm long and 1.6 mm wide up to the first bifurcation, and the leaf is a microphyll with a 43 mm long and ca. 50 mm wide blade. L/W ratio is about 1:1 and the lamina shape is ovate and symmetrical. Lamina is chartaceous. The leaflets are sessile and microphyllous and 35–43 mm long and 13–18 mm wide. Their base is acute and decurrent, whereas their lobes are obtuse and rounded. Their blades are also odd-pinnatisect with three to five alternate and asymmetrical lobes, which are obovate and asymmetrical in the middle, and increase in size acropetally. The lobes are proximally decurrent onto the primary vein, but end abruptly distally.

The overall architecture of the leaf (Fig. 5) is derived from a palmate organization with a petiole running straight and then bifurcating. The primary venation of leaflets is pinnate with a single basal vein and without naked basal veins. Agrophic veins and interior secondaries are absent. The major secondaries are craspedodromous and branch dichotomously two to three times before merging into a perimarginal vein. Rare anastomoses occur. The venation of the lobes is mainly composed of secondary veins, except for the distal lobes of each leaflet where their pinnate primary vein ends. Secondary venation is bifurcating and rather thin compared to the primary vein (0.08–0.22 mm), the width ratio secondaries ∕ primary is typically 1:2. The secondaries are regularly spaced (0.17–0.27 mm) and they form uniform moderately acute angles (30–45°) to the primary vein. The attachment of secondaries to the midvein is long decurrent, with the major ones running several millimeters subparallel to the midvein. Intersecondaries and higher order venation are absent. Vein density ranges of 4.5–4.9 (mean of 4.6 mm^−1^).

The third paratype (MB.Pb. 1997/1562) is a fruit with seeds (Fig. 3). The structure of the fruit is very similar to those of the holotype and first paratype, with 6 stipitate follicles surrounded by several innermost erect laciniate and one outermost obovate (reflexed) persistent perianth-like organs. The follicles are 24–29 mm long and 10–13 mm wide, with a 7–11 mm long stalk (Fig. 3A). The seeds are elliptical and bilaterally symmetrical, and 3–5 mm long and 2 mm wide (Fig. 3A, B). The seed coat comprises two distinct layers, with the outermost composed of elongated cells with straight to undulate walls around the micropyle but forming a jigsaw puzzle-shaped surface over the chalazal region (Fig. 3C–E). A smooth papillate or punctate wall ornamentation with a spongy appearance is also present on the outer layer, especially at the periphery of the seed. In contrast, the inner layer is composed of more or less isodiametric polygonal to irregular cells which form a reticulum, and contain spiral structures which nature remains unclear (Fig. 3D). Below the seed coat, there is no evidence of an embryo, suggesting that it is surrounded by an endosperm and that the seed is albuminous.

The following features were compiled from characters of all specimens of the protologue. The follicles have on average an estimated volume of ca. 1215 mm^3^ and display numerous transverse veins that are 0.2–1 mm apart. Each follicle contains several seeds (ca. 5–9) with on average an estimated volume of ca. 5.8 mm^3^, and arranged in a row with the micropyle facing the median longitudinal abaxial placenta.

*Santaniella acuta* Gobo, Coiffard, Bachelier, L.Kunzmann et Iannuzzi sp. nov.

#### Specific diagnosis

Leaves nanophylls and simple to microphylls with at least two leaflets. Leaflets usually sessile, with acute and cuneate base and acute and straight to convex apices. Lamina chartaceous. Fruit surrounded by obovate persistent perianth-like organs.

#### Etymology

The specific epithet refers to the distinctly acute tips of the leaflets.

#### Holotype

MB.Pb. 2000/71 (Fig. 4) (repository: Museum für Naturkunde—Leibniz Institute for Evolution and Biodiversity Science, Berlin, Germany).

### Plant Fossil Names Registry Number

PFN002913 (for new species)

#### Type horizon and locality

Open cast pit southwest of Nova Olinda, Ceará, NE Brazil. Lower Cretaceous (uppermost Barremian?/ Aptian), C6 limestone horizon, Crato Formation, Santana Group, Araripe Basin.

#### Description and remarks

The holotype (MB.Pb. 2000/71) and only specimen is a leafy axis with a fruit (Fig. 4). The axis is about 125 mm long and 2.5 mm wide. The two proximal leaves are pedately compound and of type II (Fig. 5). Their petioles are about 6–7 mm long and 1–1.6 mm wide, the leaves are microphylls with ca. 23 mm long and 15 mm wide blade. The L/W ratio is about 1.5:1 and lamina shape is ovate. Lamina is chartaceous. The leaflets are nanophyllous and 16–23 mm long and 5–6 mm wide. They are sessile, lobed to unlobed with untoothed margins, and slightly asymmetrical in the middle of the blade. Their base is acute and cuneate (asymmetrical), and their apices acute and straight to convex. In contrast, the distal-most leaves are simple and of type I (Figs. 4A, 5). One is unlobed and at least 10 mm long and 2.3 mm wide. The L/W ratio is about 4:1. The second distal leaf seems to be bilobed, but it is not well-preserved. However, we do not exclude because of their similarities that the proximal leaves were (sub)opposite and the distal leaves were a pair of bracts or bracteoles.

The overall architecture of the leaf (Fig. 5) is derived from a palmate organization with a petiole running straight and then bifurcating. The primary venation of leaflets is pinnate with a single basal vein and without naked basal veins. Agrophic veins and interior secondaries are absent. The major secondaries are craspedodromous and branch dichotomously two to three times before merging into a perimarginal vein (Fig. 4B). Rare anastomoses occur. Secondary venation is bifurcating and rather thin compared to the primary vein (0.08–0.2 mm), the width ratio between secondaries and the primary vein is typically 1:2 in the distal part of the leaflet. The secondaries are regularly spaced (0.15–0.3 mm) and they form uniform moderately acute angles (30–45°) to the primary vein. The attachment of secondaries to the midvein is long decurrent, with the major ones running several millimeters subparallel to the midvein. Intersecondaries and higher order venation are absent. Vein density is 4.5 mm^−1^.

The fruit of the holotype is an aggregate and comprises 5–6 follicles surrounded by one obovate, persistent perianth-like organ. The fruitlets are 21–25 mm long and 12 mm wide, with a 7–9 mm long stalk (Fig. 4). They have on average an estimated volume of ca. 1203 mm^3^ and display numerous transverse veins that are 0.2–0.6 mm apart. The follicles contain at least 5 seeds which are elliptical, bilaterally symmetrical, and 3–5 mm long and 2 mm wide. The seed volumes have on average ca. 5.8 mm^3^. Their follicles, seeds and perianth-like organ are similar to those of *Santaniella lobata*. However, there is no evidence of innermost laciniate, persistent perianth-like organs surrounding the follicetum in *Santaniella acuta*.

#### Cladistic analyses and potential systematics affinities

**Figure 6:**
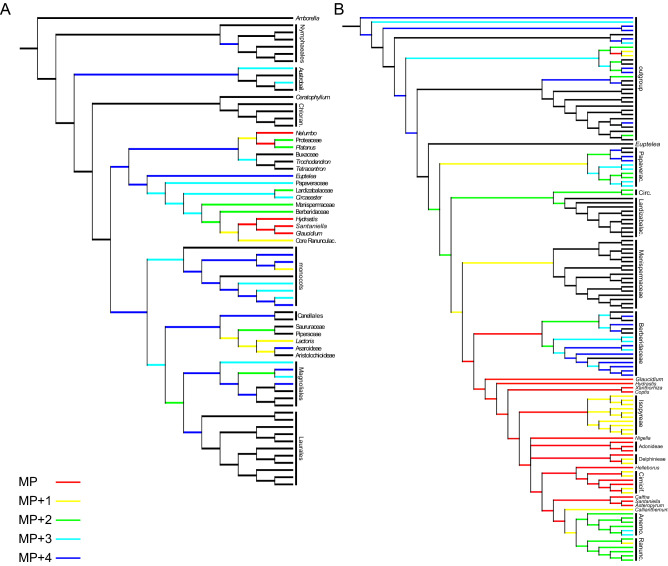
Phylogenetic position of *Santaniella* gen. nov. Most parsimonious positions of *Santaniella* after its addition to the matrix of Kvaček et al.^40^ in (**A**) and Wang et al.^45^ in (**B**). Colour scale indicates all most parsimonious positions in red (MP), and those one step less parsimonious in yellow (MP + 1), two steps less parsimonious in green (MP + 2), three steps less parsimonious in cyan (MP + 3), and four steps less parsimonious in dark blue (MP + 4) positions.

Our first cladistic analysis (Fig. 6A) was performed using the matrix by Kvaček et al.^40^ and results in five most parsimonious positions of *Santaniella* within basal eudicots, either as sister to Glaucideae (*Glaucidium*), or Hydrastideae (*Hydrastis*), or Glaucideae + Hydrastideae (successively basal-most lineages of Ranunculaceae), or sister to *Nelumbo* or Proteaceae + *Platanus* (basal eudicots). However, if *Santaniella* is sister to *Glaucidium*, it would imply changes in distichous phyllotaxis (CH22), marginal teeth (CH35) and carpel number (CH97), and as sister to *Hydrastis* changes in distichous phyllotaxis (CH22), marginal teeth (CH35) and ovule direction (CH115). Similarly, the Glaucideae + Hydrastideae position implies changes in marginal teeth (CH35), carpel number (CH97) and ovule direction (CH115). If sister to *Nelumbo*, it would imply changes in leaf dissection (CH34), ovule direction (CH115) and fruit dehiscence (CH126). Also, Nelumbonaceae differ from *Santaniella* by having large, and centrally peltate leaves, the perianth is caducous and each carpel forms a 1-seeded nutlet embedded in an enlarged and obconic receptacle^41^. Similarly, the Proteaceae + *Platanus* position would imply changes in inflorescence structure (CH42), ovule direction (CH115) and fruit dehiscence (CH126). Proteaceae also differ from *Santaniella* by having a unicarpellate gynoecium^42^, and Platanaceae by their usually distinct simple, toothed and broadly palmately lobed leaves, and long-pedunculate inflorescence with globular heads, and 1-seeded fruits interpreted as densely hairy achenes or nutlets in a globose head^41^. Inside Ranunculales, one step less parsimonious positions were recovered as sister to Ranunculaceae as a whole, or as sister to the core of the family. Other ranunculid lineages are two to four steps less parsimonious (see below the second cladistic analysis). One step less parsimonious position outside of the basal eudicots either falls within magnoliids in Piperales and would imply changes in leaf dissection (CH34), inflorescence structure (CH42), carpel number (CH97) and fruit wall (CH124), or within monocots in Dioscoreaceae, but would also imply changes in leaf dissection (CH34), inflorescence structure (CH42), carpel number (CH97) and carpel fusion (CH107). Other positions within other magnoliids or monocots are often less parsimonious and, in magnoliids, fall in lineages which often differ from *Santaniella* by the absence of several dehiscent follicular fruitlets with many seeds^2,43^ and, in the case of monocots, can be excluded by their typical linear leaves with parallelodromous or acrodromous to campylodromous venation, and sheathing bases^38^. Positions within the ANA grade are three to four steps less parsimonious, but in contrast with *Santaniella*, none of their extant lineages have plicate carpels and/or several ovules, which are synapomorphies of most mesangiosperms^44^.

Our second cladistic analysis using the dataset of Wang et al.^45^, which is more centered on Ranunculales, results in the most parsimonious positions of *Santaniella* within Ranunculales, as sister to Berberidaceae + Ranunculaceae, Berberidaceae, or Ranunculaceae (basal and core lineages), but also among basal eudicots, as sister to *Platanus* + Proteaceae (Fig. 6B). No changes in any characters are expected in all the most parsimonious positions. However, if *Santaniella* is within of crown group of Berberidaceae, it would imply changes in carpel number (CH39) and carpel form (CH40), because they fundamentally differ by their single and ascidiate carpel^43^. The most parsimonious positions within the Ranunculaceae are widespread in basal and core lineages (except Callianthemeae and the last-diverging lineages, i.e., Anemoneae and Ranunculeae). While positions inside Ranunculales (except Ranunculaceae) as sister to Menispermaceaeae or Papaveraceae would be one step less parsimonious, they would imply changes in fruit dehiscence (CH56), or in major venation (CH19), respectively. In addition, Menispermaceae also differ from *Santaniella* by having simple leaves, and the flowers gathered in inflorescences which develop fruits 1-seeded drupes^41^, and Papaveraceae by their syncarpous gynoecia with 2, rarely 3 or up to 25 carpels^43^. Two steps less parsimonious and it could be sister to Circaeasteraceae and Lardizabalaceae, and imply similar changes in major venation (CH19), and fruit dehiscence (CH56). Also, Circaeasteraceae differ from *Santaniella* by having toothed leaves, achene fruits and 1 to 3 carpels in *Circaeaster* and 5 to 9 carpels in *Kingdonia*, but with one ovule in each carpel^41,43^. In Lardizabalaceae, the flowers are gathered in inflorescences and form fleshy fruits^41^.

In summary, our cladistic analyses suggest that *Santaniella* belongs to basal eudicots, most likely to the Ranunculales or the stem lineage of Ranunculaceae. Indeed, the combination of *Santaniella* features, especially of compound leaves and a superior apocarpous gynoecia with several carpels producing many seeds, is more common in basal eudicots than in any other main lineages of angiosperms^46,47^. Also, most of traits displayed by *Santaniella* occur singularly or in various combinations in several extant members of Ranunculales (e.g., in Ranunculaceae), such as the presence of compound and lobed leaves^41^, craspedodromous secondary venation without intersecondary veins^48^, bracts (or cauline bract-like leaves) along the stem^41^, solitary and terminal flowers with short receptacles^41^, superior apocarpous gynoecium with plicate carpels with transverse veins and several ovules^43^, aggregate fruits with stalked follicular fruitlets^41^, persistent floral organs at fruiting stage^49^, and the range of size and shape of seeds^50^. Unfortunately, the nature of the persistent perianth-like organs in *Santaniella* remains unclear, and despite the above similarities the unique combination of its vegetative and reproductive traits does not fit in any extant taxon of Ranunculales.

#### Comparison to fossil record

Macro- and mesofossil records of putative basal eudicots/ranunculids are widespread in northern mid-latitudes (e.g., China^12–14^, Spain^15^, Portugal^16–18^, Russia^19,20^, Kazakhstan^21^, United States^22–24^). Combined with our findings from southern low-latitudes (Brazil), they shed light on the relatively broad paleogeographical area of this group in the Early Cretaceous.

Some taxa with ranunculidean affinities from Liaoning province, China (late Barremian to early late Aptian age) were reported by Leng and Friis^12^, Dilcher et al.^51^, and Sun et al.^13^. *Hyrcantha decussata* (Leng & Friis) Dilcher et al. shares with *Santaniella* terminal fruits with multiple ovules or young seeds, but differs by its syncarpous gynoecium with 2–4 carpels and decussate phyllotaxy of infructescences^12,51^. *Leefructus mirus* Sun et al. differs from *Santaniella* by its simple and trilobate leaves with toothed margins, and syncarpous gynoecium with 5 carpels^13^. Another eudicot from the Early Cretaceous of China, *Gansufructus saligna* B. Du., is also preserved at the fruiting stage and bears some resemblances to *Santaniella* by its persistent perianth, superior gynoecium, and multiple seeds. However, it differs in having simple leaves with poorly organized leaf venation, paniculate infructescences, and tetracarpellate syncarpous gynoecia^14^.

From the late Barremian of Spain, *Iterophyllum lobatum* Barral et al., is possibly also a member of Ranunculales and resembles *Santaniella lobata* by its lobed pinnatipartite leaves with pinnate primary and craspedodromous secondary venation. However, its leaves are always simple with a dentate margin^15^.

Charcoalified mesofossils with apocarpous gynoecia and superior ovaries containing several ovules with similar affinities to basal eudicots/Ranunculales were also described from the late Barremian to middle Albian of Portugal and the United States. However, all these fossils, e.g., *Kajanthus lusitanicus* Mendes et al.^17^, *Paisia pantoporata* Friis et al.^18^, and *Kenilanthus marylandensis* Friis et al.^24^, are only known from preanthetic or anthetic flowering material which can hardly be compared to the fruits of *Santaniella.* There is also *Teixeira lusitanica* von Balthazar et al.^16^ but it is a unisexual male flower without any evidence of carpels and therefore cannot be compared to *Santaniella*.

From Aptian to middle Albian Potomac Group sediments in the United States, putative eudicots were described as *Potomacapnos apeleutheron* Jud & Hickey, which is also characterized by lobed leaflets and veins that ramify dichotomously and fuse into a perimarginal vein like in *Santaniella*, but differs by its glandular teeth and reticulate venation^22^. The pinnately lobed leaves of *Fairlingtonia thyrsopteroides* Jud are also quite similar to those of *Santaniella lobata* with their pinnate primary veins and a craspedodromous secondary venation branching into perimarginal veins, but they have small petioles, and lobes which decrease in size acropetally and end with a glandular papillate tooth^23^.

Another potential eudicot, *Baderadea pinnatissecta* Pessoa et al., was recently described from the Crato Formation flora^6^. It consists of a single leaf with a pinnatisect dissection similar to that of the leaflets of *Santaniella lobata*, but the leaf is simple and its lobes are distinctly linear. In addition, its potential affinities with eudicots are only supported by the leaf dissection and primary venation.

#### Overall importance of our findings

Nowadays, many Ranunculales occur in areas where they could potentially undergo a fossilization process^2^. However, most of them are typically herbaceous and, in contrast to forest canopy-forming trees or shrubs, are less likely to be preserved as fossils, and also usually produce and shed less vegetative and reproductive parts over their life time^52^. In addition, they can also occur in environments characterized mostly by erosional rather than depositional processes, and thus, are even less likely to be buried within sediments^52^.

Fortunately, the depositional lacustrine environment of the Crato Formation along with the microbial mats enables the remarkable preservation potential of various organisms^53^, including herbaceous and (sub)shrub-like plants less susceptible to be preserved in the fossil record^30,54,55^. Therefore, the Crato Formation flora allows to track the evolutionary history of plant lineages that are usually not or underrepresented in the fossil record, and is also remarkable by its diversity, especially of early-diverging and basal angiosperm lineages such as Nymphaeales and magnoliids, as well as monocots and eudicots^55^. It also demonstrates that all major flowering plant lineages were already diversified and lived in low latitudes under hot and (seasonally) dry conditions by the middle Early Cretaceous.

Earlier evidence of eudicots at low paleolatitudes in the Early Cretaceous comes from tricolpate pollen^56,57^ and is now confirmed by the macrofossils described herein. These new Brazilian fossils are the most complete macrofossils of an early-diverging eudicot with both vegetative and reproductive structures strongly supporting affinities to ranunculids in Early Cretaceous tropics. With all other fossil records linked to Ranunculales from northern mid-latitudes, our results do not only support that the widespread radiation of this clade already started at this early age, but also that they were present in paleoequatorial tropical areas.

## Methods

### Fossil analysis

The material was studied using a Leica Wild M10 microscope equipped with a Leica DFC 425 camera. Macroscopic images were taken with Canon 250D camera. One specimen exhibits remaining permineralized tissues with cellular structures, especially of *in-situ* seeds, that were replaced by iron oxide. The complete fossil (MB. Pb. 1997/1562) was directly placed uncoated inside the chamber of a Zeiss EVO 50 Scanning Electron Microscope (SEM) (Carl Zeiss, Germany), and characteristics of the epidermis were observed under a low vacuum. SEM photographs were edited with Photoshop software.

### Leaf architecture

The classes of leaf size and part of leaf architecture descriptions follow Ellis et al.^58^.

### Leaf width inferences

The leaf width measurements of leaf types II (Fig. 2C) and III (Fig. 2D) were inferred based on the leaf reconstructions (Fig. 5), assuming that the leaflets were originally not overlapping. They were used to estimate the laminar size, L:W ratio, leaf shape, and medial symmetry of leaf in these specimens.

### Vein density measurements

Four lobes of *Santaniella* fossil-species (*S. lobata* = LP UFC CRT 1559a; *S. acuta* = MB. Pb. 2000/71) were used to measure the vein length per area and describe the vein density (mm of vein per mm^2^), using the program ImageJ (https://imagej.nih.gov/).

### Seed and fruitlet volume measurements

The seed and fruitlet volume were estimated based on the formula V = 4/3πab^2^, where a = L/2, b = (W + T)/4, T = 0.66 W; L = length, W = width and T = thickness, following Eriksson et al.^59^.

### Cladistic analysis

Two cladistic analyses using morphological characters of *Santaniella* were performed using first the data matrix of Kvaček et al.^40^ with all major angiosperm lineages except core eudicots, and then using the data matrix of Wang et al.^45^ which is more specific of Ranunculales. Phylogenetic analyses were conducted using Mesquite 3.61^60^, where alternative positions were explored and character states were reconstructed. Characters were unordered and equally weighted.

## Supplementary Information

Supplementary Information.
