# Supplementary material for: First evidence of ranunculids in Early Cretaceous tropics
Source: Sci Rep. 2022 Mar 23;12:5040. doi: 10.1038/s41598-022-07920-y (PMC8943169; doi:10.1038/s41598-022-07920-y)
Supplement: Supplementary file 1 — Supplementary Information. [file 41598_2022_7920_MOESM1_ESM.docx]

**Supplementary information**

**Cladistic analysis.** Two cladistic analyses using morphological characters of *Santaniella* were performed using first the data matrix of Kvacek & al. (2016) with all major angiosperm lineages except core eudicots, and then using the data matrix of Wang & al. (2009) which is more specific of Ranunculales. Phylogenetic analyses were conducted using Mesquite 3.61 (Maddison and Maddison, 2019), where alternative positions were explored and character states were reconstructed. Characters were unordered and equally weighted.

**Character coding (following Kvaček & al., 2016).** Character states coded for the fossil are in bold.

1. Habit (0) tree or shrub, **(1) rhizomatous, scandent, or acaulescent.**

22. Distichous phyllotaxis **(0) absent,** (1) on some or all branches.

25. Leaf base **(0) nonsheathing**, (1) sheathing (half or more of stem circumference).

28. Leaf blade **(0) bifacial**, (1) unifacial.

29. Leaf shape **(0) obovate to elliptical to oblong,** **(1) ovate**, (2) linear.

30. Major venation (0) pinnate with secondaries at more or less constant angle, **(1) palmate (actinodromous or acrodromous) or crowded (pinnate with crowded basal secondaries, upward decreasing angle)**, (2) parallel (lateral veins departing at low angles from the midrib and converging and fusing apically).

31. Fine venation **(0) reticulate**, (1) open dichotomous in some or all leaves.

32. Base of blade **(0) not peltate**, (1) peltate in some or all leaves.

34. Leaf dissection (0) simple, **(1) some or all leaves lobed or compound**.

35. Marginal teeth **(0) absent**, (1) chloranthoid, (2) monimioid, (3) platanoid.

42. Inflorescence **(0) solitary flower (or occasionally with 1–2 lateral flowers),** (1) botryoid, panicle, or thyrsoid (monotelic), (2) raceme, spike, or thyrse (polytelic).

43. Inflorescence partial units **(0) single flowers**, (1) cymes.

44. Inflorescence (or partial inflorescence) **(0) not modified**, (1) modified into globular head.

45. Pedicel **(0) present in some or all flowers**, (1) absent or highly reduced (flower sessile or subsessile).

49. Floral base **(0) hypanthium absent, superior ovary,** (1) hypanthium present, superior ovary, (2) partially or completely inferior ovary.

50. Floral receptacle (female portion) **(0) short**, (1) elongate.

51. Pits in receptacle bearing individual carpels **(0) absent**, (1) present.

53. Floral apex **(0) used up after production of carpels**, (1) protruding in mature flower.

54. Perianth **(0) present**, (1) absent.

61. Outermost perianth parts **(0) free**, (1) at least basally fused.

62. Calyptra derived from last one or two bracteate organs below the flower **(0) absent,** (1) present.

97. Carpel number (0) one, (1) 2–5 in one whorl or series (when phyllotaxis is spiral), (2) more than 5 in one whorl or series, **(3) more than one whorl or series.**

98. Carpel form (0) ascidiate up to stigma, (1) intermediate (both plicate and ascidiate zones present below the stigma) with ovule(s) in the ascidiate zone, **(2) completely plicate, or intermediate with some or all ovule(s) in the plicate zone.**

107. Carpel fusion **(0) apocarpous,** (1) parasyncarpous, (2) eusyncarpous (at least basally).

113. Number of ovules per carpel (0) one, (1) two or varying between one and two, **(2) more than two.**

114. Placentation **(0) ventral**, (1) laminar-diffuse or “dorsal.”

115. Ovule direction (0) pendent, **(1) horizontal**, (2) ascendent.

124. Fruit wall (0) wholly or partly fleshy, **(1) dry**.

126. Fruit dehiscence (0) indehiscent or dehiscing irregularly, dorsally only, or laterally, **(1) dehiscent ventrally or both ventrally and dorsally,** (2) horizontally dehiscent with vertical extensions.

**Character coding (following Wang & al., 2009).** Character states coded for the fossil are in bold.

1. Growth form: 0 = woody, **1 = herbaceous.**

17. Leaf position: **0 = caulescent only**, 1 = basal only, **2 = basal and caulescent**.

19. Major venation: 0 = pinnate, **1 = palmate,** 2 = dichotomous**,** 3 = parallel.

20. Blade shape: 0 = obovate to elliptical to oblong, **1 = ovate**, 2 = linear. These states were determined according to Doyle and Endress (2000).

21. Inflorescence: **0 = solitary or occasionally with one to two additional later flowers**, 1 = spike, raceme, or botryoid, 2 = richly branched (panicle or compound inflorescence or spikes, racemes, botryoids).

39. Carpel number: **0 = more than 3**, 1 = one, 2 = two, 3 = three.

40. Carpel form: 0 = ascidiate up to stigma, 1 = intermediate (both plicate and ascidiate zones present below the stigma) with ovule(s) on the ascidiate zone, **2 = completely plicate or intermediate with some or all ovule(s) on the plicate zone.**

41. Carpel fusion: **0 = apocarpous** (including pseudosyncarpous), 1 = syncarpous (including at least basally).

46. Ovule number: **0 = more than two**, 1 = one, 2 = mostly two (occasionally one or a few more than two). Ranunculaceae: Tamura (1995). These states were determined according to Doyle and Endress (2000).

55. Fruit wall: 0 = fleshy, 1 = fleshy with hard endocarp (= drupe), **2 = dry**.

56. Fruit dehiscence: **0 = dehiscent**, 1 = indehiscent.

**Supplementary figure.**

**
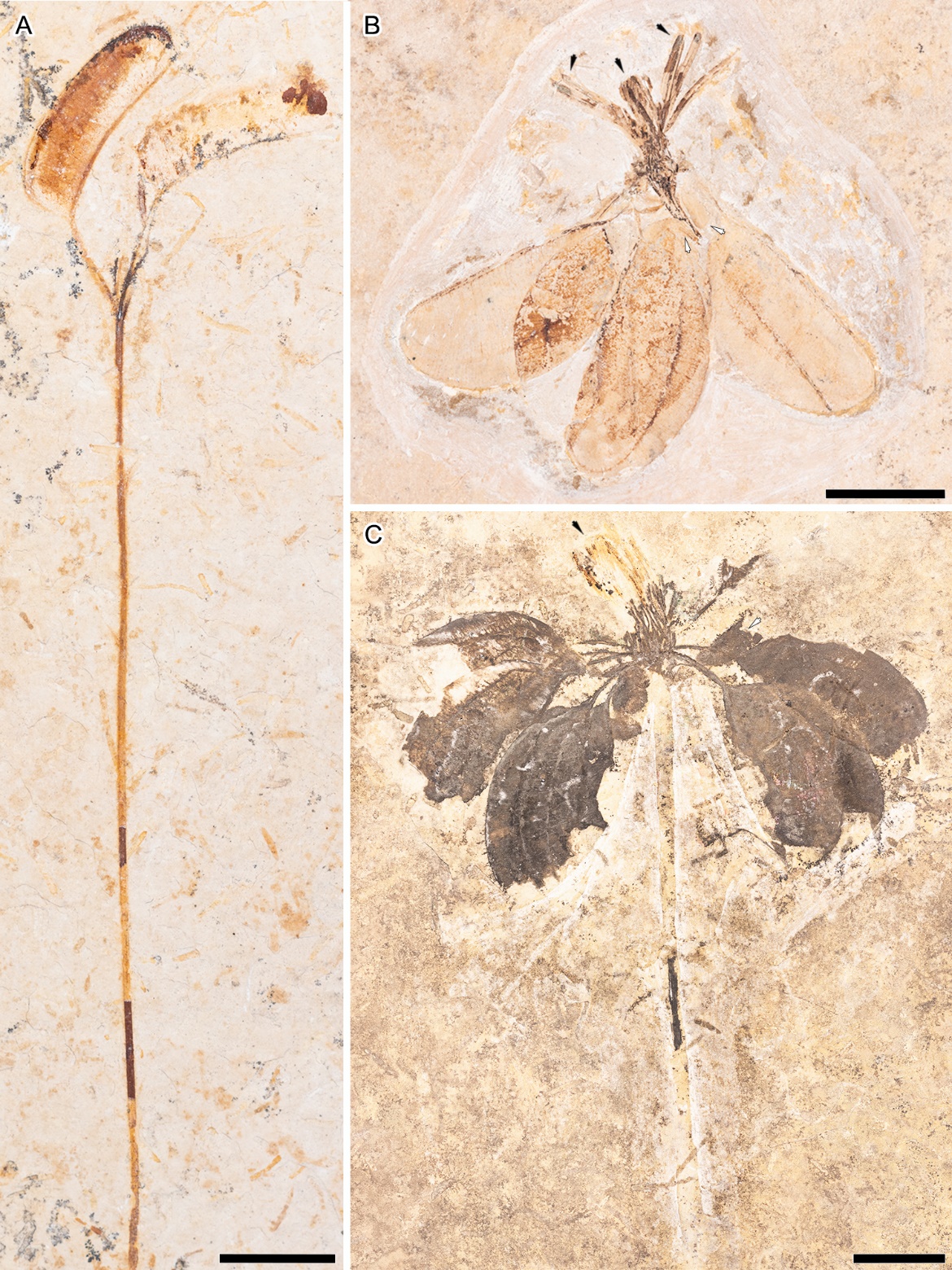
**

**Supplementary figure S1. 1. A-B,** **material with insufficient characters. A**, general habit of material (MB. Pb. 1997/1221) showing an axis attached to solitary and terminal fruit with two stipitate follicles. **B**, fruit of material (MB. Pb. 1997/1425) with two outer persistent perianth-like organs (white arrows) and several inner (black arrows) erect organs surrounding the stalks of three partly detached follicles; **C,** **paratype (MB. Pb. 1997/1348) of *Santaniella lobata* gen. et sp. nov.**, habit with a terminal aggregate fruit, with 9-10 stipitate multi-seeded follicles (white arrow points to one outer and black arrow to several inner erect and laciniate persistent perianth-like organs). Scale bars: 1cm.
